# A Rare Metastatic Site of Invasive Lobular Breast Carcinoma: A Case Report

**DOI:** 10.1155/2021/9922296

**Published:** 2021-11-22

**Authors:** Sara Husain, Mohamed Isa, Raed Almarzooq

**Affiliations:** ^1^Department of Surgery-Pediatric Surgery Unit, Salmaniya Medical Complex, Manama, Bahrain; ^2^Department of Surgery-General Surgery Unit, Consultant General Surgeon, Salmaniya Medical Complex, Manama, Bahrain; ^3^Department of Surgery-General Surgery Unit, Consultant General Surgeon and Surgical Oncologist, Salmaniya Medical Complex, Manama, Bahrain

## Abstract

Here, we report a case of a 42-year-old female patient with left lobular breast cancer-gastric metastasis (initially misdiagnosed five years ago as an invasive ductal carcinoma) presenting with dyspepsia, weight loss, and persistent vomiting lasting for four weeks. Upper GI endoscopy revealed evidence of linitis plastica, and histological and immunocytochemical analyses of the biopsy confirmed gastric metastasis secondary to invasive lobular breast carcinoma.

## 1. Introduction

Breast cancer is the most common site-specific cancer in women and the leading cause of cancer-related deaths for women aged 20 to 59 years. It accounts for 29% of all newly diagnosed cancers in women and is responsible for 14% of their cancer-related deaths [1]. Invasive lobular breast cancer accounts for 10% of all of the histological types of breast cancer and is the second most common form after invasive ductal carcinoma [2]. The most common locations of metastatic spreading for stage IV breast cancer usually include but are not limited to the lungs, spine, brain, and liver [3, 4].

Breast metastasis to the gastrointestinal tract is uncommon, occurring in 3-18% of patients at 50-78 months after the diagnosis and treatment of the primary tumor [5, 6]. In some cases, it can be the first manifestation of breast cancer metastasis. It typically presents years after the primary breast cancer has been diagnosed [7]. Gastrointestinal metastasis secondary to breast cancer is considered rare, and they occur in 3-18% of patients at 50-78 months after diagnosis and therapy of primary tumor [5, 6]. They were noted more frequently in cases of invasive lobular carcinoma than ductal carcinoma [6].

The most common gastrointestinal metastasis site is the stomach (60% of cases), followed by the esophagus (12%), colon (11%), small intestine (8%), rectum (7%), and oropharynx (1%) [1].

Most patients present with nonspecific symptoms like nausea, vomiting, dyspepsia, and weight loss, all of which can arise from other causes and be misdiagnosed as other illnesses. Therefore, many cases go unnoticed; this late presentation, late detection, and late treatment cause poor outcomes [8].

## 2. Case Presentation

**Figure 1:**
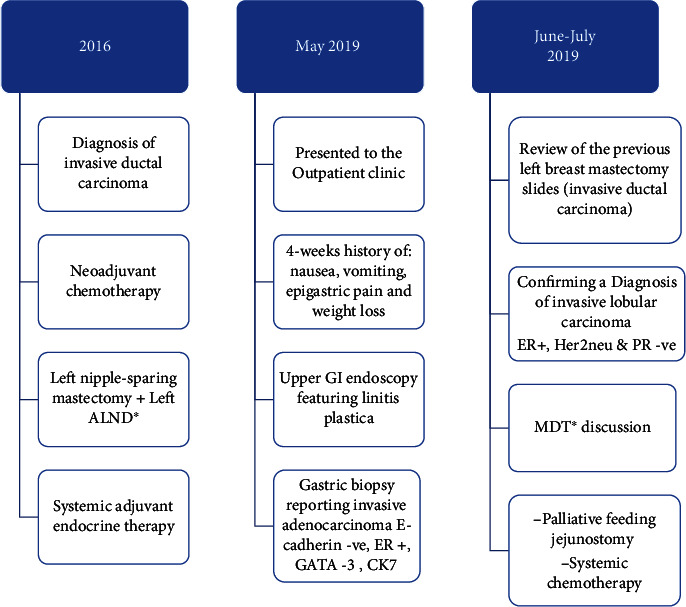
The patient's medical history timeline and clinical case presentation. ALND: axillary lymph node dissection; ER+: estrogen receptor positive; MDT: multidisciplinary team.

Here, we described the case of a 47-year-old female patient with a known case of hypothyroidism and left breast carcinoma. In early 2016, she was diagnosed with a grade 2, estrogen receptor- (ER-) positive, progesterone receptor- (PR-) positive, Her2neu-positive invasive ductal carcinoma with a TNM grade of T2N1M0 in her left breast. She underwent 14 cycles of neoadjuvant chemotherapy followed by a left nipple-sparing mastectomy with a left axillary lymph node dissection the same year. She then received adjuvant endocrine therapy (Figure 1).

She presented to our outpatient clinic in May 2019 with a 4-week history of nausea, vomiting, epigastric pain, and weight loss. She was seen initially by a gastroenterologist who performed an upper GI endoscopy that indicated signs of linitis plastica, which was biopsied and histopathologically assessed.

**Figure 2:**
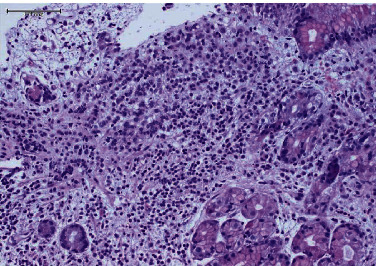
High power of the gastric biopsy with expanded lamina propria by malignant cells arranged in cords.

**Figure 3:**
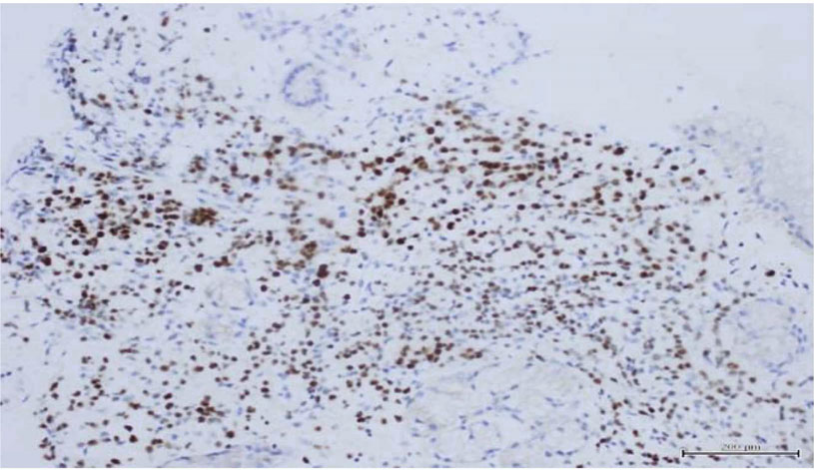
ER highlights the malignant cells, found in the gastric biopsy slides.

Histopathological analysis of the stomach biopsy sections (Figures 2 and 3) showed gastric mucosa infiltrated by neoplastic cells. The neoplastic cells were arranged in sheets of single pleomorphic cells with small, round nuclei, and inconspicuous nucleoli. Immunohistochemical analysis revealed the neoplastic cells were positive for GATA-3 and ER and negative for PR and Her2-neu. The proliferative marker (Ki-67) was detected in 40% of the cells. E-cadherin, GCDFP-15, and CDX2 were all negative, while CK7 was positive. The biopsy taken from the esophageal mucosa exhibited neither dysplasia nor malignancy.

Given the gastric biopsy results and our mounting suspicion that the metastasis originated from a lobular, rather than ductal, breast carcinoma, we reviewed the slides containing the left breast tissue collected during the patient's mastectomy. This histological assessment revealed a relatively well-defined tumor composed of sheets of neoplastic cells. The neoplastic cells were pleomorphic with round nuclei, small nucleoli, intracytoplasmic lumina, and signet-like morphology. No in situ components or ducts were observed. E-cadherin was mostly negative with focal cytoplasmic staining and P120 had cytoplasmic immunoreactivity, which were both repeated twice.

Based on the immunohistochemical comparison between the gastric and breast tumor slides, the patient was diagnosed with metastatic invasive gastric adenocarcinoma (Lauren diffuse type) originating from a lobular rather than ductal breast carcinoma.

**Figure 4:**
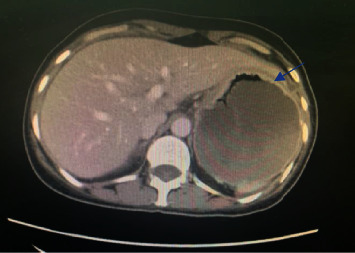
An image of the CT scan of the abdomen and pelvis showing a diffuse gastric wall thickening, about 17 mm (blue arrow).

Further imaging studies were performed to rule out other possible metastases and nodal involvement. A CT scan of the abdomen and pelvis (Figure 4) revealed a significant suspicious circumferential wall thickening that involved the pylorus and extended up to the second part of the duodenum. This caused a distention of the stomach and distal esophagus, leading to gastric outlet obstruction. However, neither nodal involvement nor further metastases were detected.

At this point, the case was discussed by the multidisciplinary national tumor board. As a result, the patient underwent palliative feeding jejunostomy insertion. Intraoperatively, a small nodule in the omentum was found incidentally, which was resected and sent for histopathological analysis.

Histopathological examination of the omentum specimen revealed focal, hard, tiny nodules on serial sections. Moreover, the omentum contained infiltrating foci of small, uniform malignant cells in the trabeculae with an Indian-file growth pattern, some of which had intracytoplasmic lumina.

Immunohistochemically, the tumor cells exhibited GATA-3 positivity, E-cadherin focal membranous and cytoplasmic (aberrant) expression, and P120-cytoplasmic positivity. Based on these findings, our final report concluded that the metastasis originated from a lobular carcinoma, which aligned with the patient's known clinical history (Table 1).

The patient is currently being treated at a specialized oncology center with a systemic chemotherapy regimen.

## 3. Discussion

In the case described here, 5 years after her initial primary breast cancer-invasive ductal carcinoma diagnosis was made, the patient presented with signs and symptoms of gastric outlet obstruction. An initial, month-long misdiagnosis of acute gastritis complicated and prolongated the diagnostic timeline. The histopathology and immunohistochemistry of the gastric biopsy contained features of adenocarcinoma with no E-cadherin expression, indicative of gastric metastasis, rather than a primary gastric tumor relevant to the patient's history of breast cancer. A CT scan of the abdomen and pelvis revealed features of gastric outlet obstruction and thickening of the stomach wall with no obvious primary etiology.

With the high suspicion index of metastasis originating from a lobular, rather than a ductal breast cancer based on the findings of the gastric biopsy, the patient's previous specimen slides of the left breast mastectomy were reviewed again, The microscopic assessment revealed a relatively well-defined tumor consisting of sheets of neoplastic cells. The neoplastic cells are pleomorphic with round nuclei, small nucleoli, intracytoplasmic lumina, and signet-like morphology. No in situ components or duct formation was seen. E-cadherin is mostly negative with focal cytoplasmic staining, and P120 shows cytoplasmic staining.

Based on the microscopic and immunohistochemical comparison between the gastric and the breast tumor slides, the final histopathological assessment concluded a metastatic invasive gastric adenocarcinoma (Lauren: diffuse type, originating from a breast lobular carcinoma rather than invasive breast ductal carcinoma).

In cases of invasive lobular carcinoma, the diagnosis of gastric metastasis is challenging as it is quite rare and most of the symptoms are nonspecific and mimic those of a primary gastric malignancy. In 97% of cases, gastric metastases from breast cancer are derived from invasive lobular carcinoma. In those cases, a histopathological comparison of the original primary breast carcinoma biopsy slides is essential to differentiate gastric metastasis from primary gastric carcinoma [9, 10]. Thorough investigations are required to differentiate between the two pathologies.

Diagnosing primary and metastatic gastric cancers solely on gross endoscopy results is challenging. As gastric metastases are mostly localized to the submucosal and seromuscular layers, the endoscopy results can appear normal in up to 50% of the cases [10]. Hence, endoscopy alone is insufficient for diagnosing gastric metastasis.

Histological assessment can be helpful as some morphological differences do exist between these two forms of gastric cancer. While both contain signet ring cells, primary gastric signet ring cells have a multivacuolated cytoplasm, while lobular metastatic cells have a well-defined univaculoated cytoplasm that is somehow specific [11, 12]. For cases where these morphological distinctions are unclear, a detailed immunohistochemical analysis is essential. Connel et al. [13] demonstrated the utility of immunohistochemistry in distinguishing between gastric metastases and breast cancer and primary gastric cancer. A comparison between the two concluded that ER, PR, GCDP, CK5/6, CK5/6, CK20, MUC5AC, MUC6, DAS-1, and CDX2 faithfully distinguish primary gastric carcinomas from metastatic breast carcinomas. Of these, ER, PR, and GCDFP are highly specific to metastatic breast carcinomas, whereas CK20, DAS-1, MUC2, MUC5AC, MUC6, and CDX2 are highly specific to primary gastric carcinomas [13, 14].

Interestingly, the expression levels of ER, PR, GCDFP, and CK5/6 are increased in gastric metastasis originating from the breast. GCDFP, ER, and PR were not expressed and 14% for CK5/6 in gastric metastases from primary gastric cancer. The expression levels of ER were 72%, 78% for GCDFP-15, 61% for CK5/6, and 33% for progesterone receptor in metastatic cancer; thus, ER, PR, and GCDFP-15 are crucial in the differentiation of gastric metastasis vs. primary gastric cancer [13, 14].

Other markers, like keratin 20 (K 20), are expressed in gastric, pancreatic, colorectal, and transitional cell carcinomas, while it is not observed in any breast cancer [12, 15, 16]. CK7 in contrast found in 90% of carcinomas of the breast and its expression was also observed in 50–64% of primary gastric adenocarcinomas [12, 15, 16]. Therefore, CK7 and CK20 expression patterns are useful in characterizing metastatic lesions of uncertain origin. About 30% of gastric adenocarcinomas have a CK7+/CK20+ profile, 20% are CK7–/CK20+, 10% are CK7–/CK20–, and only 20% are CK7+/CK20– [12, 15, 16].

So, detailed immunohistochemical analysis is essential for any final, definitive differentiation between these two cancers. In this case, the immunohistochemical analysis of the stomach biopsy showed that the neoplastic cells were positive for GATA-3 and ER and negative for PR and Her2-neu. E-cadherin, gross cystic disease fluid protein 15 (GCDFP-15), and CDX2 were all negative, while CK7 was positive. The proliferative marker (Ki-67) was 40%.

Other investigations may include an abdominal CT scan or an endoscopic ultrasound, which can help to support the diagnosis by revealing a thickened gastric wall or further metastases in other locations. However, the most effective means of reaching a definitive diagnosis and differentiating primary gastric cancer from gastric metastases originating from breast cancer is a detailed immunohistochemical analysis [17].

## 4. Conclusions

Gastric metastases from invasive lobular carcinomas are uncommon. It is important to a keep high index of suspicion when treating patients who present with nonspecific gastrointestinal symptoms with a history of invasive lobular breast carcinoma, as this may be indicative of gastric metastasis. Immunohistochemistry analysis is the only diagnostic modality there is to differentiate between primary gastric cancer and gastric metastasis.

## Figures and Tables

**Table 1:** Immunoprofile comparison of the tumor cells in the primary vs. metastatic tumor.

IHC marker	Breast tumor cells	Metastatic cells from the stomach specimen
ER	Positive	Positive
PR	Positive	Negative
Her-2	Positive	Negative
E-cadherin	Negative	Negative
